# Isolated gastrointestinal hereditary angioedema associated with a pathogenic *SERPING1* variant arising *de novo*: a case report

**DOI:** 10.3389/fmed.2026.1936529

**Published:** 2026-09-03

**Authors:** Ziyu Yuan, Weipeng Lin, Yue Zheng, Xizi Yuan, Ming Luo, Lingyu Fu, Haiyan Zhang

**Affiliations:** 1 The Second Clinical College of Guangzhou University of Chinese Medicine, Guangzhou, China; 2 The First Clinical College of Guangzhou University of Chinese Medicine, Guangzhou, China; 3 Department of Gastroenterology, The Second Affiliated Hospital of Guangzhou University of Chinese Medicine, Guangzhou, China

**Keywords:** abdominal pain, *de novo*, diagnostic delay, hereditary angioedema, isolated gastrointestinal angioedema, *SERPING1*

## Abstract

Hereditary angioedema (HAE) is a rare genetic disorder typically characterized by cutaneous and submucosal edema. Cases presenting exclusively with gastrointestinal symptoms, devoid of concurrent or historical cutaneous or upper airway manifestations, are uncommon. This atypical presentation poses a considerable diagnostic challenge and often leads to a marked delay in diagnosis, particularly in patients without a family history. We report the case of a 28-year-old female with an over 6-year history of recurrent, self-limiting abdominal pain. During a severe attack, abdominal computed tomography angiography (CTA) revealed jejunal wall edema and ascites, accompanied by a dramatic surge in D-dimer (>20.00 mg/L FEU) and fibrin degradation products (FDP >120.00 mg/L), closely mimicking mesenteric ischemia. However, a retrospective review of medical history identified the persistent consumption of complement C4 (0.03–0.05 g/L). Subsequent testing showed a functional C1 esterase inhibitor (fC1-INH) level of <7.0% and a C1 esterase inhibitor (C1-INH) antigen level of 6.81 μg/mL, confirming type 1 HAE. Whole-exome sequencing (WES) identified the nonsense variant NM_000062.3:c.990C>A, p.(Tyr330Ter), in *SERPING1*. The variant was not detected in either biological parent, supporting a *de novo* origin. Under the American College of Medical Genetics and Genomics (ACMG) and the Association for Molecular Pathology (AMP) framework, it was classified as pathogenic on the basis of PVS1 and PS2. An abdominal attack in May 2025 resolved rapidly after icatibant administration. Long-term prophylaxis (LTP) with lanadelumab was subsequently initiated following an individualized shared decision-making process. No breakthrough attacks or treatment related adverse events occurred from June 2025 through June 2026. This report highlights that HAE should be considered in patients with recurrent, unexplained, self-limiting abdominal pain, and C4 should be included in the initial evaluation. A negative family history does not exclude HAE.

## 1 Introduction

Hereditary angioedema (HAE) is a rare autosomal dominant disorder characterized by recurrent, self-limiting episodes of cutaneous and submucosal edema, most commonly affecting the skin, abdomen, and upper airway (1). The vast majority of cases are caused by pathogenic variants in *SERPING1*, which encodes the C1 esterase inhibitor (C1-INH) (2). Although gastrointestinal symptoms occur during attacks in up to 70% of HAE patients, cases restricted to gastrointestinal involvement, with no previous or concurrent cutaneous or upper airway edema, are uncommon (3, 4). Such presentations are diagnostically challenging, particularly when the family history is negative. Such atypical presentations are highly susceptible to being confused with conditions like surgical acute abdomen or inflammatory bowel disease (IBD), predisposing patients to severe diagnostic delays, inappropriate treatments, and the risks of unnecessary surgical interventions (5).

We describe a case of HAE associated with a *SERPING1* variant that arose *de novo*. The patient presented exclusively with recurrent severe abdominal pain over a 6-year period. This case highlights the differential diagnostic reasoning for such atypical HAE.

## 2 Case report

A 28-year-old female was admitted to our hospital with a more than 6-year history of recurrent, self-limiting abdominal pain, occasionally accompanied by nausea and vomiting during severe episodes. The patient denied any history of rash, skin swelling, or dyspnea either during the attacks or previously. She had no significant past medical history of chronic diseases, surgeries, or long-term medication use, and her family history was negative for similar symptoms or genetic disorders.

Since her initial onset in December 2017, the patient had experienced multiple misdiagnoses. During her third attack in 2023, a contrast-enhanced CT scan revealed swelling of the small bowel wall and mesentery with ascites, and a colonoscopy indicated ileal ulcers. Although her complement C4 level was significantly reduced (0.05 g/L; reference range: 0.1–0.4 g/L), this crucial clue was overlooked, and she was treated symptomatically for “ileal ulcers.” During her fourth attack in July 2024, a CT scan showed thickening and edema of the ileal wall and dilation of the proximal small bowel. An external hospital diagnosed her with “incomplete intestinal obstruction” and recommended exploratory laparotomy, which the patient fortunately declined.

In August 2024, the patient was admitted to our institution. A comprehensive serological workup targeting autoimmune and vasculitic etiologies (including antinuclear antibodies, extractable nuclear antigen antibodies and antineutrophil cytoplasmic antibodies) was unremarkable, effectively excluding systemic lupus erythematosus (SLE) and associated mesenteric vasculitis. Testing for *Clostridioides difficile* antigen was positive, but the toxin assay was negative, and the absence of diarrhea did not support a related infection. Computed tomography enterography (CTE) demonstrated mild mural thickening of the rectum, colon, and terminal ileum. Enteroscopy and multi-site intestinal mucosal biopsies revealed only non-specific chronic inflammation, excluding Crohn’s disease, intestinal Behçet’s disease, and intestinal lymphoma. Additionally, an interferon-gamma release assay for *Mycobacterium tuberculosis* was positive; however, the absence of systemic tuberculosis symptoms (e.g., low-grade fever, night sweats), combined with the CTE and enteroscopy findings, excluded intestinal tuberculosis.

**Figure 1:**
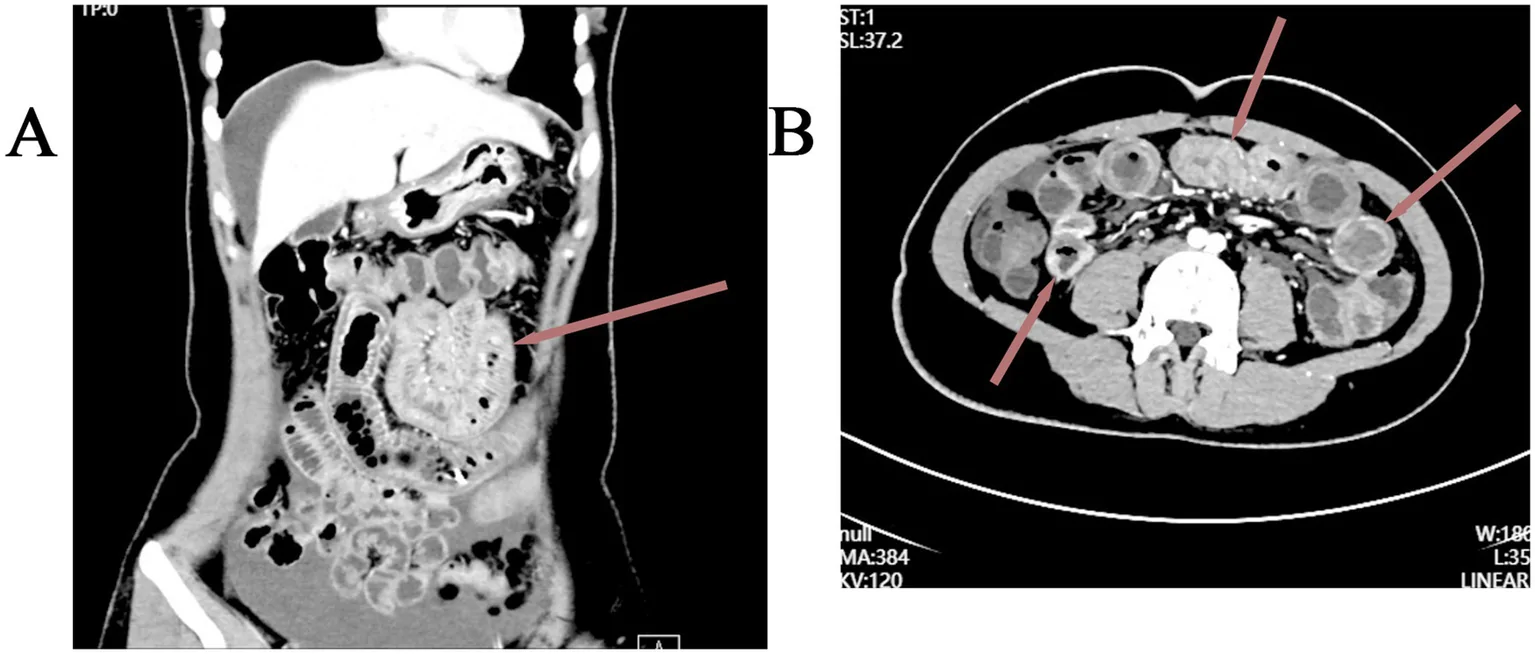
On September 3, 2024, during an exacerbation of abdominal pain, abdominal computed tomography angiography (CTA) revealed diffuse thickening and edema of the jejunal wall compared with previous scans, along with new-onset abdominopelvic fluid (**A**, **B**).

On September 3, 2024, the patient’s abdominal pain worsened. Laboratory tests revealed an abnormal surge in D-dimer (>20.00 mg/L FEU; reference: <0.50 mg/L FEU) and fibrin degradation products (FDP > 120.00 mg/L; reference: <5.00 mg/L). An emergent abdominal computed tomography angiography (CTA) ruled out mesenteric thrombosis but revealed diffuse thickening and edema of the jejunal wall compared to previous scans, along with new-onset abdominopelvic fluid [Figure 1]. Re-evaluating the medical history, we identified a critical diagnostic breakthrough: a persistent decline in C4 levels, dropping from 0.05 g/L in December 2023 to 0.04 g/L at early admission, and further to 0.03 g/L during this acute pain crisis. The combination of recurrent self-limiting abdominal pain, transient mural edema with ascites, and a persistent decrease in C4 strongly pointed toward HAE. Subsequent laboratory testing confirmed the diagnosis of type I HAE: functional C1 esterase inhibitor (fC1-INH) was <7.0% (reference: ≥58.9%), C1-INH antigen level was 6.81 μg/mL (reference: 81.46–291.29 μg/mL), and C4 concentration was profoundly low at 0.020 g/L (reference: 0.073–0.373 g/L).

**Figure 2:**
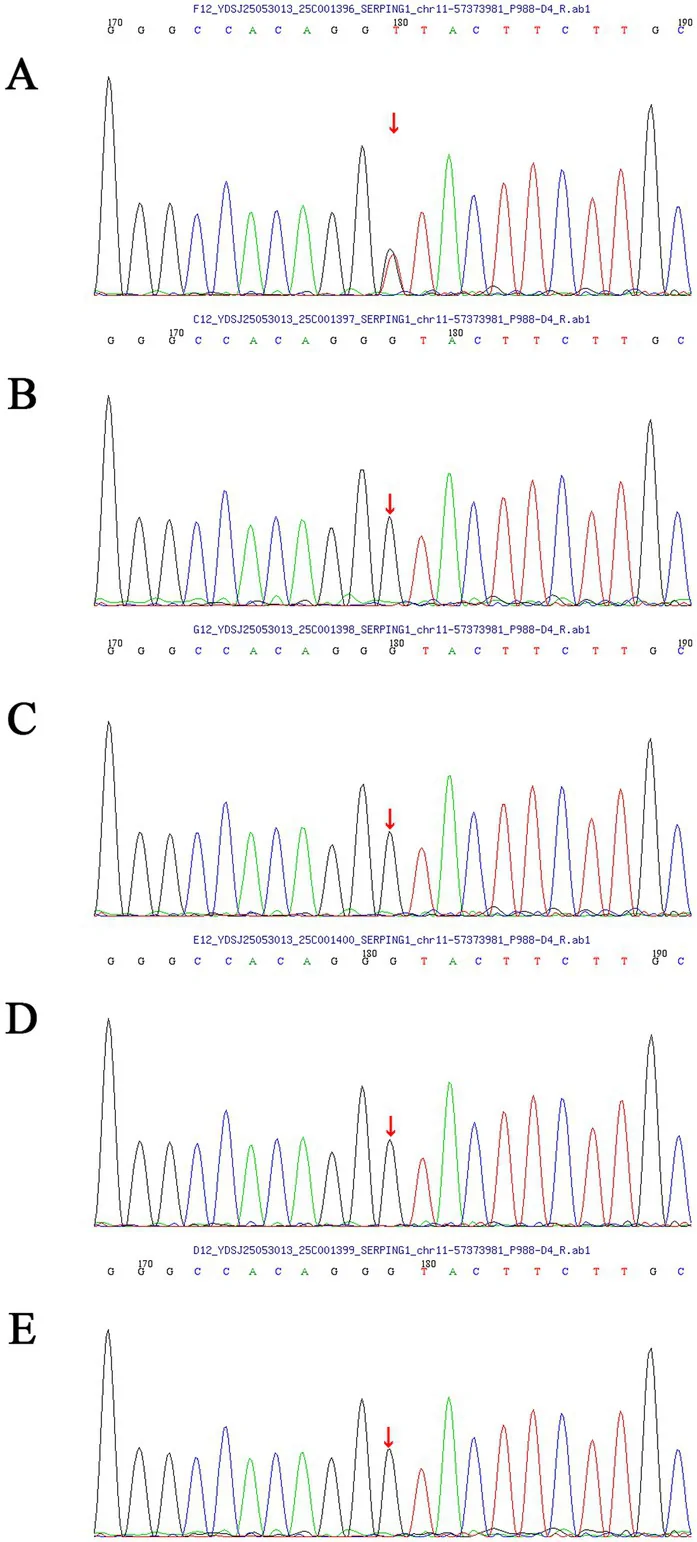
Gene sequence analysis report. Panel **A** shows a heterozygous variant in the SERPING1 gene of the patient, while Panels **B**, **C**, **D**, and **E** demonstrate no abnormalities in the SERPING1 gene of the patient’s parents and siblings. Gene sequence of the SERPING1 gene located on chromosome 11q12-q13.1. The standard nomenclature for this variant is NM_000062.3:c.990C>A (coding region variant), with the corresponding protein-level alteration designated as p.(Tyr330Ter).

Whole-exome sequencing (WES) identified a heterozygous nonsense variant in the *SERPING1* [NM_000062.3:c.990C>A, p.(Tyr330Ter)] (6). This variant had previously been reported by Ponard et al. in a study of HAE (7), it cannot be classified as a novel variant. Pedigree testing did not detect the variant in either parent or in the patient’s siblings, supporting a *de novo* origin [Figure 2]. Available medical records also documented fC1-INH, C1-INH antigen levels, and C4 within the respective reference ranges in her parents and siblings. According to the American College of Medical Genetics and Genomics (ACMG) and the Association for Molecular Pathology (AMP) framework, the variant was classified as pathogenic based on PVS1 and PS2. PVS1 was applied because this nonsense variant is expected to cause loss of *SERPING1* function. PS2 was applied because the variant was not detected in either parent, supporting *de novo* occurrence (6, 8).

During another acute abdominal pain attack in May 2025, the patient achieved rapid and complete symptom resolution following a subcutaneous injection of 30 mg icatibant. Although her attack frequency was low, long-term prophylaxis (LTP) was considered because of marked anticipatory anxiety and the considerable travel distance between her home and the treating hospital. Following an individualized shared decision-making process that also took her financial circumstances and insurance coverage into account, lanadelumab was initiated at a dose of 300 mg subcutaneously every 2 weeks, while icatibant remained available for on-demand treatment (ODT). No breakthrough attacks or treatment related adverse events were recorded from June 2025 through June 2026.

The timeline of the patient’s clinical event, key diagnostic evaluations, and clinical management are summarized in Table 1.

**Table 1:** The timeline of the patient’s clinical event, key diagnostic evaluations, and clinical management.

Timepoint and clinical events	Key diagnostic workup and findings	Therapeutic interventions and diagnosis
Dec 2017: initial episode	Previous medical records were unavailable due to document loss.	Symptomatic treatment administered; suspected diagnosis of SLE-associated enteritis.
2018: second episode	Comprehensive initial workup revealed no specific diagnostic abnormalities.	Anti-inflammatory and analgesic symptomatic treatments administered; diagnosis remained unclear.
Dec 2023: third episode	C4 significantly decreased (0.05 g/L); contrast-enhanced CT showed edema of the small bowel wall and mesentery; colonoscopy revealed ileal ulcers.	Gastroprotective and supportive therapies administered; misdiagnosed as “ileal ulcer” (the critical clue of decreased C4 was overlooked).
Jul 2024: fourth episode	CT scan indicated thickening and edema of the ileal wall, accompanied by proximal small bowel dilation.	Conservative management including anti-infective and gastroprotective therapies administered; misdiagnosed as “incomplete intestinal obstruction”; laparoscopic exploration was recommended but refused by the patient.
Aug–Sep 2, 2024: fifth episode	CTE showed mild mural thickening of the rectum, colon, and terminal ileum. Enteroscopy and multi-site intestinal mucosal biopsies indicated non-specific chronic inflammation.	Analgesic and supportive care administered; certain differential diagnoses were ruled out, but the underlying etiology remained a significant diagnostic challenge.
Sep 3, 2024: critical turning point	D-dimer > 20.00 mg/L FEU and FDP > 120.00 mg/L. C4: 0.03 g/L. Abdominal CTA ruled out mesenteric thrombosis, revealing diffusely thickened and edematous jejunal walls compared to prior scans, along with new-onset abdominopelvic ascites.	High suspicion of HAE based on the clinical characteristics of recurrent self-limiting abdominal pain, transient mural edema with ascites, and persistently low C4 levels.
Sep 10, 2024: definitive diagnosis	fC1-INH < 7.0%, C1-INH antigen level: 6.81 μg/mL, C4: 0.01974 g/L. Genetic testing via WES identified a heterozygous nonsense mutation in the *SERPING1* gene [c.990C > A (p. Tyr330Ter)].	Confirmed diagnosis of Type I HAE.
May 2025: acute relapse post-diagnosis	No specific investigations performed.	Rapid and complete symptom resolution following on-demand subcutaneous injection of icatibant 30 mg during the acute phase. Subsequently, long-term prophylaxis was initiated with subcutaneous lanadelumab 300 mg every 2 weeks; the patient has remained relapse-free since.

## 3 Discussion

The central pathophysiologic mechanism of HAE is quantitative deficiency or functional impairment of C1-INH caused by pathogenic *SERPING1* variants, which in turn drives unregulated plasma kallikrein activity, excessive bradykinin generation, aberrantly increased vascular permeability, and ultimately recurrent angioedema (1, 2). When this edema occurs in the gastrointestinal tract, bradykinin binds to endothelial B2 receptors, causing massive fluid extravasation into the submucosal layer of the bowel wall, clinically manifesting as self-limiting abdominal pain and ascites (9). Symptoms typically peak within the first 24 h and gradually subside over the subsequent 48 to 72 h, leaving no permanent structural tissue damage once the edema resolves (10). This clinical course perfectly aligns with our patient’s presentation.

During the acute attack, the patient exhibited an abnormal spike in D-dimer and FDP. This occurs because the unchecked activation of the kallikrein-kinin system during an acute HAE attack is intrinsically coupled with the concurrent activation of the intrinsic coagulation pathway and the secondary fibrinolytic system, leading to significantly elevated D-dimer and FDP levels (11, 12). Such manifestations of a hypercoagulable state can easily mislead clinicians to suspect fatal surgical acute abdomens, such as mesenteric thrombosis or intestinal ischemia, further compounding diagnostic confusion. Recognizing this mechanistic link is essential for avoiding misdiagnosis and iatrogenic trauma.

Detecting the persistent decline in C4 was the most critical breakthrough in screening for HAE and resolving this prolonged diagnostic odyssey. Reports indicate that 81–96% of HAE patients exhibit a persistent reduction in C4 (13). In this case, significant C4 abnormalities were documented during previous hospitalizations but were consistently overlooked, leading to missed opportunities for early diagnosis and treatment. Therefore, when encountering patients with recurrent, unexplained abdominal pain combined with transient bowel wall edema, routine screening for C4 is imperative. Once C4 is found to be decreased, further testing for fC1-INH and C1-INH antigen levels is mandatory, supplemented by genetic testing when necessary. It is worth noting that since C4 levels may normalize in some patients during attack-free intervals or following attenuated androgen therapy, a normal C4 level alone should not easily rule out HAE (4). Moreover, while the majority of patients have a positive family history, our patient’s family history was negative, which was a major reason why early clinicians failed to prioritize HAE in their differential diagnosis. Consequently, a negative family history is insufficient to exclude HAE, as approximately 25% of HAE cases arise from *de novo SERPING1* variants (14). Furthermore, due to the estrogen-dependent nature of disease severity, incomplete penetrance in male carriers can mask the familial inheritance pattern, resulting in a false-negative family history (15).

Because the pathogenesis of HAE is mediated by bradykinin rather than histamine release or cellular immune infiltration, conventional emergency medications, including antihistamines, glucocorticoids, and epinephrine, are notoriously ineffective (16). In recent years, the management paradigm for HAE has advanced significantly toward targeted biologic therapies (17). Given her low attack frequency, ODT with icatibant was a reasonable option (10). LTP with lanadelumab was initiated following individualized shared decision making that considered her anticipatory anxiety, the considerable distance between her home and the treating hospital, and the affordability of treatment under her insurance coverage. No breakthrough attack or treatment related adverse event occurred from June 2025 through June 2026. These observations do not establish that the same approach is appropriate for other patients or health care systems. Given the low baseline attack frequency, the attack-free follow-up period cannot be confidently attributed to LTP. Moreover, no formal cost-effectiveness analysis was conducted.

This report has several limitations. First, it is a retrospective case report. Information regarding the frequency, severity, duration, and potential triggers of some early symptoms was based on the patient’s recollection and may therefore be subject to recall bias. Second, available medical records stated that C4, fC1-INH, and C1-INH antigen results were within reference ranges in the patient’s parents and siblings. The original laboratory reports had been lost, so the exact values could not be independently verified. Third, failure to detect the variant in peripheral blood from either parent supports a *de novo* origin but does not fully exclude low level parental somatic mosaicism or germline mosaicism (18). Fourth, no cutaneous or upper airway edema had been documented by June 2026, but the HAE phenotype may evolve over time. Future extragastrointestinal manifestations cannot be excluded, and the effectiveness of LTP cannot yet be determined (10). Therefore, the term “isolated gastrointestinal” applies only to the pre-LTP observation period. Fifth, the feasibility of extending the lanadelumab dosing interval, discontinuing LTP, or using ODT alone was not formally assessed. With icatibant available for ODT, the indication, dosing interval, treatment burden, and continuing need for LTP should be reassessed regularly.

## 4 Conclusion

HAE confined to the gastrointestinal tract is readily missed, particularly when the family history is negative, and may be mistaken for a surgical acute abdomen. In patients with recurrent, unexplained, self-limiting abdominal pain, clinicians should measure C4 and include HAE in the differential diagnosis.

## Data Availability

The datasets presented in this article are not readily available because they contain potentially identifiable clinical and genetic information from a single patient and are subject to ethical and privacy considerations. Requests to access the datasets should be directed to the corresponding authors, Lingyu Fu (18813966752@163.com) and Haiyan Zhang (zhanghaiyan128@126.com).
